# Murine Neural Stem/Progenitor Cells Protect Neurons against Ischemia by HIF-1α–Regulated VEGF Signaling

**DOI:** 10.1371/journal.pone.0009767

**Published:** 2010-03-22

**Authors:** Kate M. Harms, Lu Li, Lee Anna Cunningham

**Affiliations:** Department of Neurosciences, University of New Mexico Health Sciences Center, Albuquerque, New Mexico, United States of America; Universidade Federal do Rio de Janeiro (UFRJ), Brazil

## Abstract

Focal cerebral ischemia following middle cerebral artery occlusion (MCAO) stimulates a robust cytogenic response from the adult subventricular zone (SVZ) that includes massive proliferation of neural stem/progenitor cells (NSPCs) and cellular migration into the injury area. To begin to explore beneficial roles of NSPCs in this response, we investigated the ability of embryonic and postnatal NSPCs to promote neuronal survival under conditions of *in vivo* and *in vitro* ischemia. Intracerebral transplantation of NSPCs attenuated neuronal apoptosis in response to focal ischemia induced by transient MCAO, and prevented neuronal cell death of cortical neurons in response to oxygen-glucose deprivation (OGD) in culture. NSPC-mediated neuroprotection was blocked by the pharmacological inhibitors of vascular endothelial growth factor (VEGF), SU1498 and Flt-1Fc. Embryonic and postnatal NSPCs were both intrinsically resistant to brief OGD exposure, and constitutively expressed both hypoxia-inducible factor 1α (HIF-1α) transcription factor and its downstream target, VEGF. Genomic deletion of HIF-1α by Cre-mediated excision of exon 2 in NSPC cultures resulted in >50% reduction of VEGF production and ablation of NSPC-mediated neuroprotection. These findings indicate that NSPCs promote neuronal survival under ischemic conditions via HIF-1α-VEGF signaling pathways and support a role for NSPCs in promotion of neuronal survival following stroke.

## Introduction

Two predominant neurogenic regions persist within the adult mammalian brain, located within the subgranular zone (SGZ) of the hippocampal dentate gyrus and the subventricular zone (SVZ) surrounding the lateral ventricles [1]–[4]. Neural stem/progenitor cells (NSPCs) that reside within these brain regions are mitotically active, self-renewing cells with the potential to differentiate into neurons, oligodendrocytes, or astrocytes [2], [5]–[7].

Following focal cerebral ischemia, neural stem/progenitor cells (NSPCs) in the SVZ proliferate and migrate to the lesioned site in both rodents and humans [8]–[14]. Migration of NSPCs and their progeny may be critical for post-ischemic repair, since ablation of progenitor proliferation leads to increased infarct volume [15] and the time course of this migratory response occurs concomitant with partial spontaneous recovery of motor deficits [16], [17]. Although hundreds of thousands of cells emigrate from the SVZ into the injured striatal parenchyma, the number of neuroblasts that survive long-term represents <0.2% of striatal neurons lost to ischemic injury [18]. That such a low number of new neurons contribute significantly to the reconstruction of striatal circuitry seems unlikely. Nevertheless, evidence suggests that SVZ derivatives migrating into the injured striatal parenchyma contribute importantly to the process of endogenous wound healing following ischemic insult, through processes apart or in addition to neuronal replacement [19]. Additional mechanisms include stabilization of nascent vasculature following stroke-induced angiogenesis and/or prevention of progressive neuronal cell loss via release of angiogenic and neurotrophic factors.

Vascular endothelial growth factor (VEGF) is the most well-studied angiogenic factor, but also has been shown to exert direct neurotrophic signaling and to promote adult neurogenesis [20]–[22]. The most potent inducer of VEGF gene expression is hypoxia [23]. VEGF is transcriptionally regulated by hypoxia inducible factor-1α (HIF-1α), which translocates to the nucleus following hypoxia-induced stabilization, dimerizes with HIF-1β (ARNT), and activates the hypoxia response element (HRE) in the promoter region of the VEGF gene (for review see [24]). VEGF is also regulated by HIF-independent mechanisms including other transcription factors and co-activators [25] (for reviews see [26], [27]), micro-RNAs [28], and inflammatory mediators [29]. In neurons, VEGF mediates its neurotrophic effects via the receptor tyrosine kinase, VEGFR-2 (Flk-1/KDR) [21]. Following stroke, exogenous administration of VEGF has been shown to reduce infarct size and improve neurological performance [30]. These effects are thought to be due to both a direct neurotrophic action of VEGF on neurons and stimulation of angiogenesis for re-establishment of blood flow following ischemic damage. Transgenic mice with VEGF-overexpression also display increased neurogenesis, decreased infarct volume, and improved motor function [31].

Neural stem cells have previously been shown to provide neuroprotection when transplanted into the adult brain in rodent models of stroke [32]–[34], raising the possibility that the endogenous SVZ response to stroke may also provide neuroprotection of penumbral neurons at risk of delayed cell death. We have previously demonstrated that embryonic neural stem cells secrete diffusible VEGF, which underlies their ability to protect endothelial cells against severe ischemia and promote angiogenesis in ischemic striatum [35]. In the present study, we investigated whether embryonic or postnatal NSPCs also provide neuroprotection against cerebral ischemia utilizing both *in vitro* and *in vivo* approaches. Here, we demonstrate that both embryonic and postnatal NSPCs are robustly resistant to oxygen-glucose deprivation (OGD), and provide neuroprotection under ischemic conditions, an effect that is mediated by HIF-1α-regulated release of diffusible VEGF by primary NSPCs. These studies have important implications for both the therapeutic use of exogenous NSPCs in stroke and also suggest a potential neuroprotective role for endogenous NSPCs in adult brain repair.

## Results

### NSPCs provide neuroprotection against focal ischemia *in vivo*

**Figure 1:**
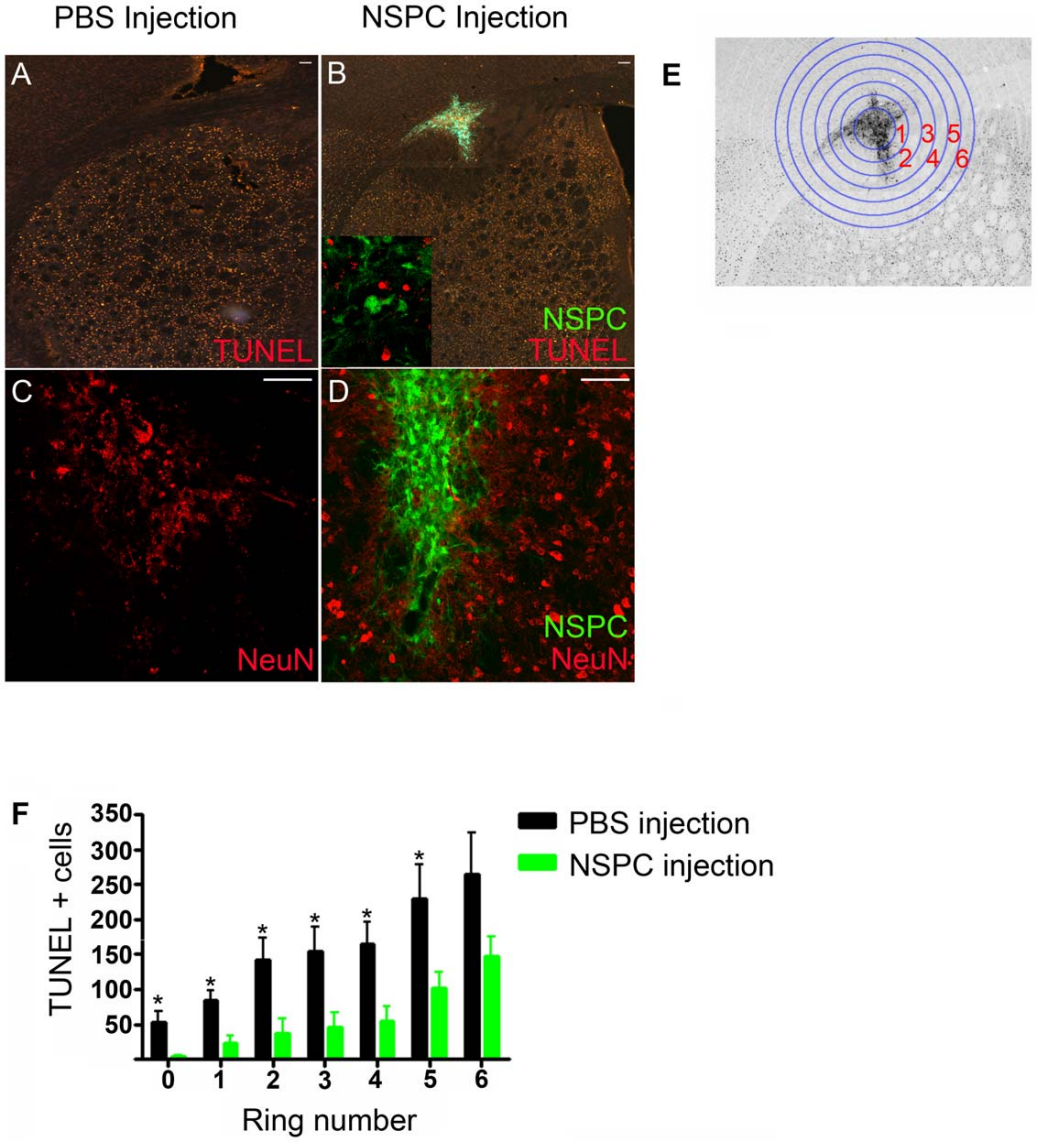
NSPCs protect against 30 minute MCAO. (**A–D**) Coronal histological sections through the ischemic striatum 3 days following MCAO, stained for TUNEL (A,B) or NeuN (C,D). Mice received intrastriatal injections of exogenous PBS (A,C) or EGFP^+^NSPCs (B,D,E) 72 hr prior to MCAO. Inset in (B) shows 40× magnified view of the injection site. (**E**) Monochrome conversion of image shown in B to demonstrate concentric ring structures used for Sholl analysis. (**F**) Quantification of TUNEL+ cells using Sholl analysis performed on fluorescent images. *p<0.05, n = 5 mice per group. Scale bar: A,B  =  20 µm; C,D  =  10 µm.

To determine whether mouse NSPCs are neuroprotective in our animal model of focal ischemia, we isolated embryonic NSPCs from transgenic mice that express enhanced green fluorescent protein (EGFP) under the β-actin promoter. We have previously demonstrated that these NSPCs fulfill the criteria of multi-potentiality and self-renewal [35]. EGFP-NSPCs were expanded in culture and subsequently transplanted into the right dorsal striatum of adult recipient mice. Control mice received vehicle injections of phosphate buffered saline only. Seventy-two hours following transplantation, recipients were subjected to an ipsilateral transient middle cerebral artery occlusion (MCAO) for 30 minutes, followed by three days of reperfusion. As shown in Figure 1A, MCAO resulted in a robust apoptotic response at three days as assessed by TUNEL labeling of injured striatum in vehicle treated mice. EGFP-NSPC recipients displayed >50% fewer TUNEL+ nuclei compared to vehicle controls (Figure 1B), particularly in the area immediately surrounding the graft and extending out to at least 4.5 mm from the graft border (Figure 1E,F). Conversely, healthy appearing NeuN^+^ neuronal nuclei were numerous near the graft, whereas control mice displayed widespread loss of NeuN^+^ cells (Figure 1C,D). These data demonstrate robust NSPC-mediated neuroprotection against focal ischemia in the MCAO model. We previously reported that NSPC-transplant promotes vascularization and proliferation of blood vessels within the ischemic striatum [35], suggesting an effect of NSPCs on the vasculature. Activated neutrophils, as identified by myeloperoxidase (MPO) staining, were not observed in either vehicle- or NSPC-injected striatum, suggesting that NSPC transplantation does not alter the inflammatory response following stroke (Figure S1). To test whether NSPCs have a direct effect on neurons during ischemia, we shifted to an *in vitro* cell culture system using a well-characterized OGD model [36].

### NSPCs provide neuroprotection against OGD and are intrinsically resistant to *in vitro* ischemia

**Figure 2:**
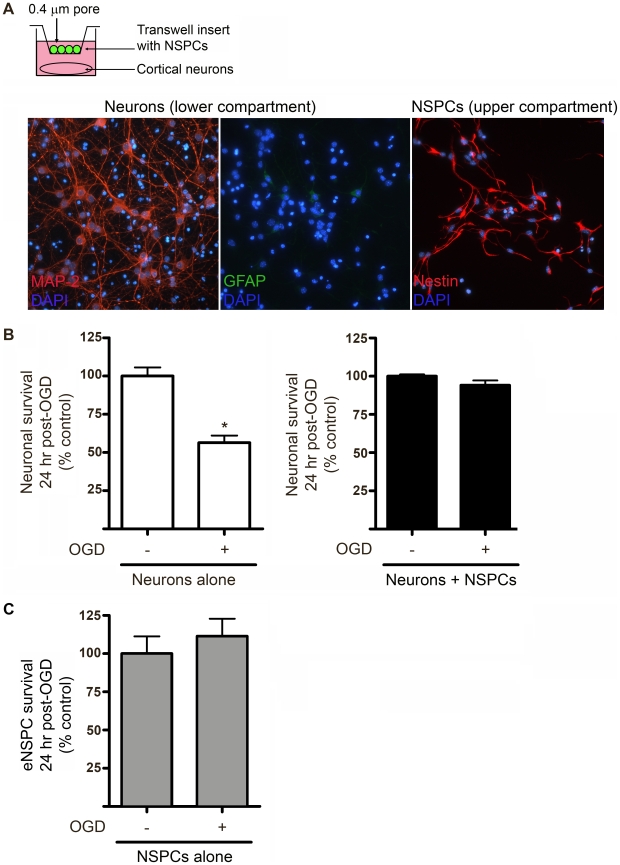
NSPCs provide neuroprotection against OGD and are resistant to *in vitro* ischemia. (**A**) Diagram of transwell coculture. Micrograph depicts cortical neurons in lower compartment immunofluorescently labeled for MAP-2 (red) and GFAP (green). NSPCs in upper compartment are immunofluorescent for nestin (red). Nuclei are labeled with DAPI (blue). (**B**) Neuronal survival at 24 hrs following 2 hr exposure to OGD in the absence (left) or presence (right) of NSPCs. (**C**) Survival of NSPCs grown as monocultures in upper compartment. Data were acquired using the MTT colorimetric viability assay as described in methods section. *p<0.01, Student's t-test, n = 4 cultures/group.

We utilized a transwell coculture paradigm to begin to delineate the mechanisms underlying NSPC-mediated neuroprotection. NSPCs and cortical neurons were cultured separately as monocultures or were cocultured in separate compartments of porous transwells (0.4 µm pore size), which allowed for sharing of conditioned media but did not allow cell-cell contact between NSPCs and neurons seeded in the upper *vs.* lower compartments, respectively. As shown in Figure 2, cortical cultures contained MAP-2^+^ neurons, with minimal contamination by GFAP^+^ astrocytes, and NSPC cultures were uniformly immunopositive for the stem cell marker, nestin. Neuronal and NSPC cultures were subjected to control or OGD conditions for 2 hours and assessed for cell viability 24 hr later using an MTT assay. As shown in Figure 2B, cortical cultures underwent approximately 40% cell loss within 24 hr following OGD, as previously described [37], but only ∼12% cell loss when cocultured with NSPCs. Importantly, NSPC monocultures were robustly resistant to 2 hour OGD and displayed no significant cell loss at 24 hours (Figure 2C). These data indicate that NSPCs provide robust neuroprotection of cortical neurons in culture via diffusible substance(s), and are themselves resistant to OGD conditions.

### Diffusible VEGF is responsible for NSPC-mediated neuroprotection against OGD

**Figure 3:**
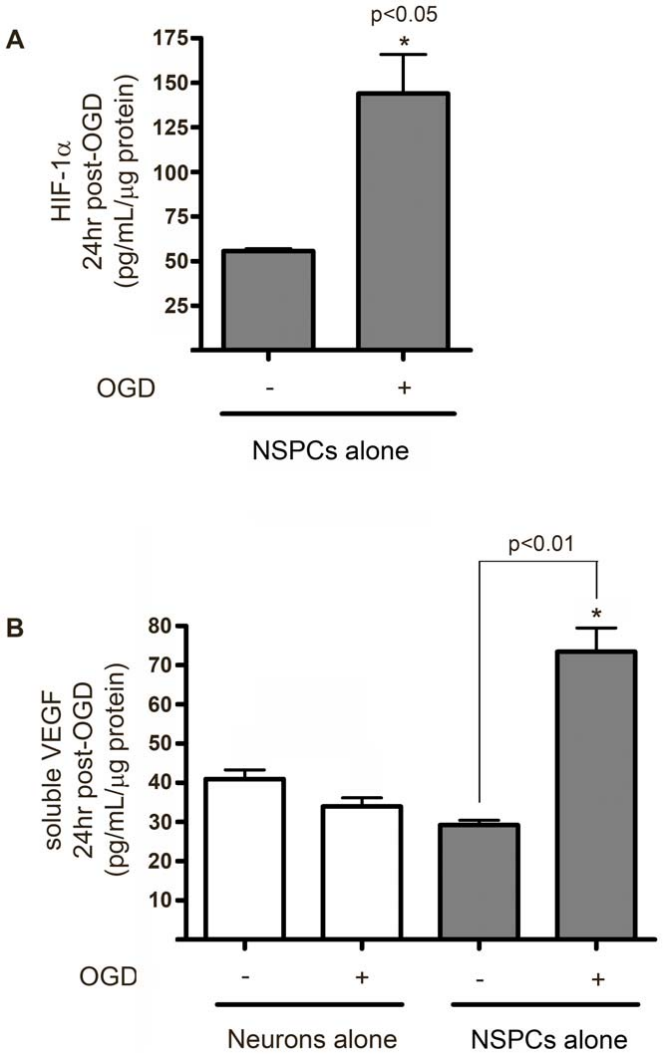
HIF-1α and VEGF expression in eNSPC cultures under control *vs.* OGD conditions. (**A**) HIF-1α protein levels in cell lysates from eNSPCs at 24 hrs following a 2 hr exposure to OGD or control conditions. (**B**) VEGF within neuronal or NSPC conditioned media following 2 hr exposure to control or OGD conditions. Media was conditioned for 24 hrs following exposure to OGD or control conditions. *p<0.05, Student's t-test, n = 4 cultures/group.

HIF-1α is a key mediator of the adaptive cellular response to hypoxia and acts through transcriptional and non-transcriptional pathways to regulate the activities of genes involved in cell survival and angiogenesis, including VEGF. As shown in Figure 3A, NSPC monocultures constitutively expressed HIF-1α and displayed a nearly 3-fold increase in HIF-1α expression measured 24 hr following a 2 hr exposure to OGD. In contrast, HIF-1α was not measurable in neuronal monocultures under nonhypoxic conditions or at 24 hr following a 2 hr OGD exposure (not shown). As shown in Figure 3B, both neuronal and NSPC monocultures constitutively expressed the HIF-1α target gene, VEGF, and released VEGF into the culture media. VEGF release was not statistically different in cortical monocultures following OGD, but was increased 2.5-fold in NSPC monocultures as measured 24 hr following 2 hr exposure to OGD. Culture media from NSPCs was also used to measure the presence of brain-derived neurotrophic factor (BDNF), however BDNF was undetected in both control and OGD-exposed NSPC media (data not shown).

**Figure 4:**
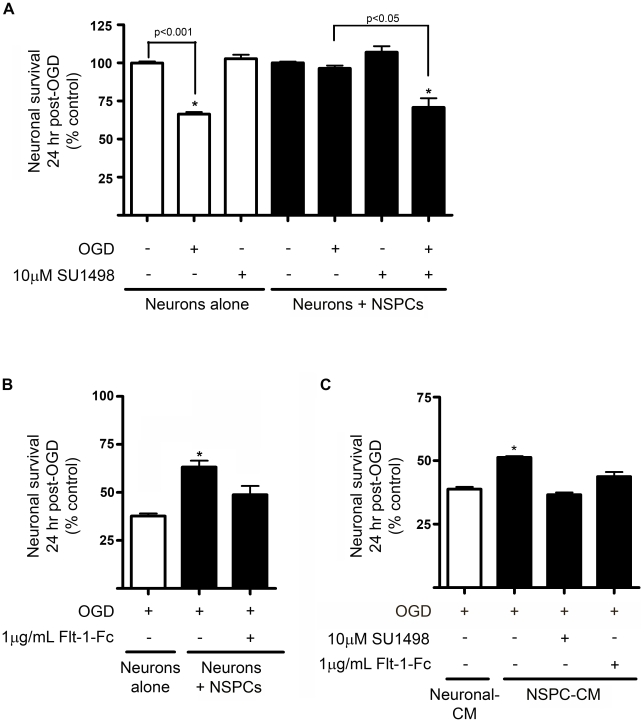
Pharmacological inhibition of VEGF signaling impairs the neuroprotective effects of eNSPCs. (**A,B**) Neuronal viability 24 hrs following exposure to 2 hr OGD vs. control conditions in the presence or absence of NSPCs and the VEGFR kinase inhibitor, SU1498 (A) or the VEGF receptor decoy receptor, Flt-1-Fc (B). (**C**) Neuronal viability in the presence or absence of NSPC-conditioned media and SU1498 or Flt-1Fc. *p<0.05, n = 4 cultures/group.

To determine whether diffusible VEGF is responsible for NSPC-mediated neuroprotection, neurons in monoculture or in transwell coculture with NSPCs were exposed to OGD in the presence or absence of the VEGFR2 kinase inhibitor, SU1498 (10 µM; Figure 4A) or the VEGF decoy receptor, Flt-1-Fc (1.0 µg/ml; Figure 4B) added at the onset of OGD and present for 24 hr thereafter. As demonstrated in Figure 4A, SU1498 had no effect on neuronal viability under non-ischemic conditions, but completely inhibited the ability of NSPCs to provide neuroprotection against OGD in coculture. Prevention of VEGF-VEGFR interaction at the cell surface with Flt-1-Fc also impaired the neuroprotection provided by cocultured NSPCs (Figure 4B). Dose-response analysis for both SU1498 and Flt-1-Fc showed that neither compound was cytotoxic to neurons in monoculture at the concentration used in this study (Figure S2). Exposure of neuronal cultures to NSPC-conditioned media resulted in significant neuroprotection against OGD, an effect that was also blocked by SU1498 and Flt-1-Fc inhibition of VEGF signaling (Figure 4C). Flt-1-Fc was not cytotoxic to NSPCs, and had no effect on the ability of NSPCs to withstand OGD conditions (Figure S3). These data indicate that NSPC-mediated neuroprotection is mediated by diffusible VEGF and dependent on neuronal VEGFR signaling.

### Gene deletion of HIF-1α impairs NSPC-mediated neuroprotection

**Figure 5:**
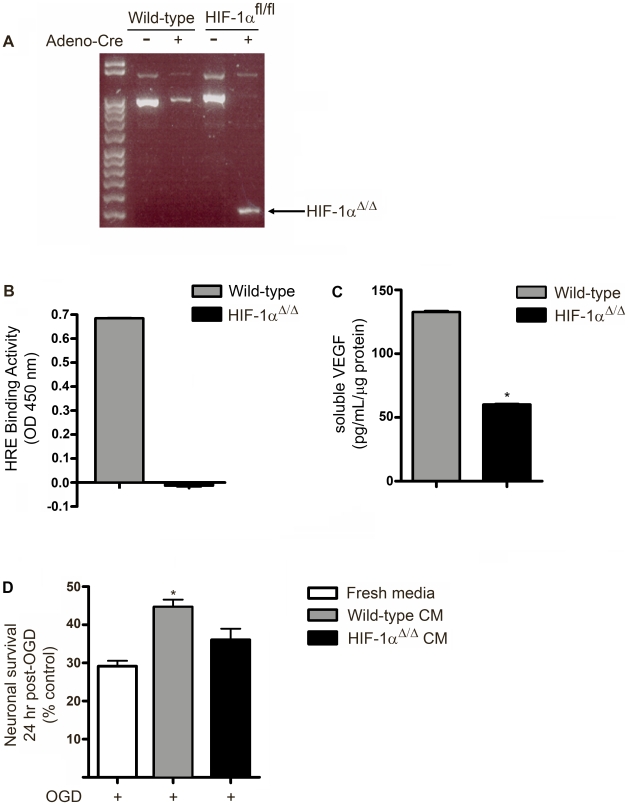
Gene deletion of HIF-1α impairs neuroprotective ability of eNSPC. (**A**) PCR amplification products from genomic DNA isolated from wild-type and HIF-1α^fl/fl^ NSPCs exposed to 50 MOI Ad-CMV-Cre for 48 hrs. Excision of exon 2 from HIF-1α gene is indicated by the shorter band at 250 bp, present after Ad-CMV-Cre exposure of HIF-1α ^fl/fl^ but not wild-type NSPCs. (**B**) Transcriptional activity of HIF-1α in wild-type vs. HIF-1α^Δ/Δ^ NSPCs assayed by HRE binding assay. (**C**) VEGF in 24 hr conditioned media from wild-type vs. HIF-1α^Δ/Δ^ NSPCs. (**D**) Viability of cortical cultures 24 hr following 2 hr OGD in the presence or absence of wild-type *vs.* HIF-1α^Δ/Δ^ NSPCs CM. *p<0.05, n =  4 cultures/group.

To determine the extent to which endogenous NSPC HIF-1α governs VEGF expression and NSPC-mediated neuroprotection, eNSPCs were isolated from transgenic mice that harbor loxP sites flanking exon 2 of the HIF-1α gene at both alleles (HIF-1α^fl/fl^) [38]. HIF-1α^wt/wt^ and HIF-1α^fl/fl^ NSPC cultures were incubated for 24 hr with adenovirus harboring a bacterial Cre-recombinase gene sequence to confer Cre expression (Ad-CMV-Cre). As shown in Figure 5A, transduction of NSPC cultures with Ad-CMV-Cre resulted in excision of HIF-1α exon 2 in HIF-1α^fl/fl^ NSPCs (HIF-1α^Δ/Δ^), but not in HIF-1α^wt/wt^ NSPCs, as indicated by PCR analysis of genomic DNA isolated 24 hr following removal of Ad-CMV-Cre. HIF-1α transcriptional activity was completely abolished as assessed by the inability of nuclear extract proteins to bind the hypoxia response element (HRE) in HIF-1α^Δ/Δ^ cells, but not in HIF-1α^wt/wt^ cells (Figure 5B). HIF-1α gene deletion in NSPCs also resulted in a 50% reduction of VEGF release by HIF-1α^Δ/Δ^ NSPCs compared to HIF-1α^wt/wt^ NSPCs in response to anoxia (Figure 5C). HIF-1α gene deletion had no effect on NSPC survival at 24 hr following 2 hr OGD (96.8 %±8.5 % *vs.* 101.6 %±6.7 %, HIF-1α^wt/wt^ *vs.* HIF-1α^Δ/Δ^, respectively, ns, n = 4; Figure S4), but abrogated the neuroprotective effects of NSPC conditioned media (Figure 5D). Thus, HIF-1α appears to be necessary for NSPC-mediated neuroprotection since media from HIF-1α^Δ/Δ^ cultures failed to protect neurons from OGD-induced death.

### Postnatal NSPCs are neuroprotective, resistant to ischemia and constitutively express stabilized HIF-1α and VEGF

**Figure 6:**
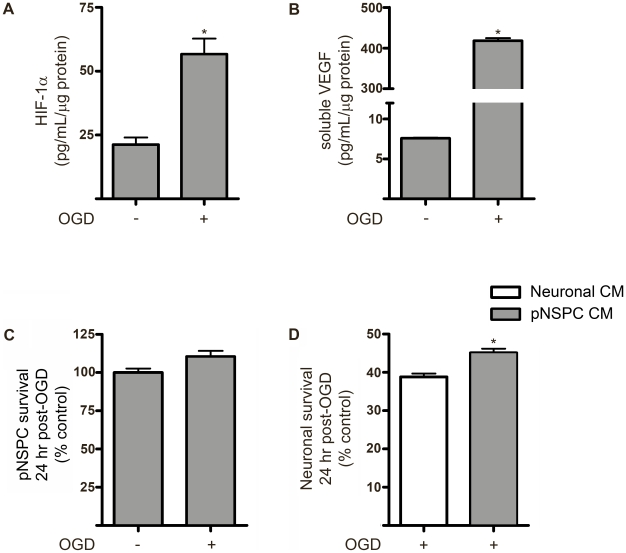
Postnatal NSPCs are neuroprotective and resistant to ischemia. (**A**) HIF-1α protein levels in cell lysates and (**B**) VEGF concentration in conditioned medium from pNSPCs at 24 hr following 2 hr exposure to control or OGD conditions. (**C**) Viability of pNSPCs at 24 hr post-OGD. (**D**) Viability of cortical neuronal cultures 24 hr following 2 hr OGD in the presence of neuronal *vs.* pNSPC conditioned medium. *p<0.05, n = 4 cultures/group.

To determine whether postnatal NSPCs also confer neuroprotection and are resistance to *in vitro* ischemia, we isolated NSPCs from postnatal day 28 mouse SVZ and expanded the pNSPCs in culture using the same paradigm as for eNSPCs. As shown in Figure 6 (A and B), pNSPCs constitutively expressed stabilized HIF-1α and VEGF at levels similar to eNSPCs under non-ischemic conditions, with robust induction of both proteins in response to 24 hr anoxia. pNSPCs also displayed resistance to 2 hr OGD exposure (Figure 6C), and media conditioned by pNSPCs provided neuroprotection against 2 hr OGD (Figure 6D).

## Discussion

In this study, we investigated the neuroprotective effects of embryonic and postnatal NSPCs under conditions of ischemia, and the role of HIF-1α-regulated VEGF signaling in this process. We found that exogenous NSPCs protect neurons against focal ischemic injury induced by MCAO *in vivo*, and protect embryonic cortical neurons against OGD exposure in culture. In contrast to primary cortical neurons, NSPCs display intrinsic resistance to brief 2 hr OGD exposure in culture, and constitutively express stabilized HIF-1α and its downstream target, VEGF. Pharmacological blockade of VEGF signaling in culture impairs neuroprotection, and HIF-1α gene deletion in NSPCs results in both impaired VEGF production and impaired neuroprotection against OGD. These observations provide evidence that NSPCs promote neuronal survival under conditions of ischemia via HIF-1α-regulated VEGF signaling and suggest that NSPCs may play a neuroprotective role following stroke.

VEGF is recognized as both a vasculotrophic factor and a neurotrophic factor. VEGF expression in the brain is downregulated in early postnatal life after formation of the cerebral vasculature is complete, but becomes upregulated in adult brain in response to stroke or mechanical injury [39]–[41]. Administration of exogenous VEGF following stroke results in reduced neuronal cell death, increased angiogenesis and increased vascular permeability [30], [42], [43] (reviewed in [42], [44], [45]). VEGF also exerts a direct neuroprotective effect against *in vitro* ischemia, mediated by neuronal VEGFR2 signaling and downstream activation of the phosphatidylinositol 3-kinase (PI3 kinase)/Akt pathway in cultured neurons [46]–[51]. Our studies suggest that VEGF release by NSPCs underlies *in vitro* neuroprotection against ischemia, via direct activation of neuronal VEGFR2 receptors, since neuroprotection was abrogated by the presence of the VEGFR2 kinase inhibitor, SU1498, and by the presence of the VEGFR decoy receptor, Flt-1-Fc. In conjunction with our previous report [35], VEGF could have a dual effect in the graft: improved revascularization and direct neuroprotection.

VEGF production by both embryonic and adult NSPCs in culture has previously been reported [35], [52]–[54]. NSPC expression of VEGF is known to be upregulated in response to hypoxic and metabolic stress [35], [54]. Our present study demonstrates that cortical cultures also release diffusible VEGF under normoxic conditions but do not display increased VEGF production at 24 hr following 2 hr OGD, whereas VEGF release by NSPCs is increased 2–3 fold within 24 hr post-OGD. Although inhibition of VEGFR2 signaling blocks NSPC-mediated neuroprotection, inhibition of VEGFR2 signaling has no impact on the survival of neurons under normoxic conditions, and has no effect on the ability of NSPCs to survive ischemic insult. These observations suggest that increased VEGF production by NSPCs underlies neuronal protection against *in vitro* ischemia, but does not mediate NSPC resistance to ischemic conditions and is not required for survival of cortical neurons under normoxic conditions.

One of the most potent regulators of VEGF expression is HIF-1α. HIF-1α is primarily regulated at the post-translational level, with protein stability dependent upon pO_2_. In most mammalian cell types, HIF-1α protein is hydroxylated under normoxic conditions, binds the von Hippel-Lindau (VHL) protein, and is rapidly degraded by the 26S proteosome. Under conditions of hypoxia, HIF-1α is not hydroxylated and rapidly accumulates to enter the nucleus and dimerize with HIF-1β (ARNT), forming the HIF-1 transcriptional complex that regulates target genes through cis-acting hypoxia response elements (HREs). In most cell types HIF-1α mRNA is constitutively transcribed and translated, but the protein only has a half-life of less than 5 minutes under non-hypoxic conditions (for review, see [24], [55]). Our observation that HIF-1α is constitutively stabilized by cultured NSPCs, even at 21% O_2_, suggests that NSPCs may also utilize a non-oxygen dependent mechanism for maintaining HIF-1α stability and that HIF-1α plays a fundamental role in NSPC homeostasis under both normoxic and hypoxic conditions. Previous studies have demonstrated O_2_-independent stabilization of HIF-1α by heat shock protein 90 (HSP-90) through binding the PAS domain of HIF-1α [56]. HSP-90 has also been shown to be involved in the regulation of HIF-1α stability in embryonic mesencephalic NSPCs [57].

While the role of constitutive HIF-1α stabilization in NSPCs is not completely understood, HIF-1α is recognized as a multifunctional protein that regulates the expression and activity of multiple genes involved in angiogenesis, glucose metabolism, proliferation and self-renewal, apoptosis, and migration through both transcriptional and non-transcriptional signaling pathways [58]-[61]. Others have shown that upregulation of HIF-1α in neural progenitor cells enhances their ability to survive *in vitro* and following transplantation [62], as well as provide neuroprotection against ischemia [32]. In addition to VEGF, HIF-1α is a potent inducer of erythropoietin (EPO), which promotes neurogenesis, NSPC proliferation and neuroprotection following ischemia [58], [63]–[65]. However, neural-specific EPOR knockdown does not increase infarct volume following focal cerebral ischemia and therefore endogenous levels of EPO may not provide neuroprotection following stroke [65]. Conversely, antisense blockade of VEGF *in vivo* results in an enlargement of infarct volume caused by transient MCAO [66]. In the present study, we found that HIF-1α gene deletion results in >50% reduction of VEGF production and a concomitant impairment of neuroprotective activity in NSPC-conditioned medium. However, these data also indicate that VEGF production is at least partially regulated by HIF-1α-independent mechanisms in NSPCs, since HIF-1α gene deletion did not completely block VEGF production.

We previously demonstrated that embryonic NSPCs protect endothelial cells against ischemia-induced damage and stimulate increased endothelial cell proliferation via HIF-1α-regulated VEGF signaling mechanisms [35]. Here, we extend those studies to demonstrate that NSPCs also provide neuroprotection through similar signaling mechanisms. While neural stem cells were initially recognized as potential therapeutic agents for stroke and neurodegenerative disease based on their potential to replace lost neurons and integrate into host circuitry, our studies support the possibility that the therapeutic potential of neural stem cells may lie predominantly in their ability to promote endogenous brain repair processes. Following stroke, NSPCs and their progeny migrate to the lesioned area and inhibition of this migration leads to an increase in infarct size [15]. Our study reveals that NSPCs may directly protect against ischemic injury through the release of diffusible VEGF. Further studies will require NSPC-targeted gene deletion of VEGF and HIF-1α to delineate the role of endogenous NSPCs in neuroprotection *in vivo*, as well as their effects on other cell types within the neurovascular niche. While *in vitro* modeling is crucial to understanding the molecular mechanisms of the interactions between neurons and NSPCs, *in vivo* studies will account for the ischemic microenvironment.

While our studies suggest that NSPCs themselves are neuroprotective and important for vascular remodeling following stroke, care must be taken with development of therapeutical strategies. Direct NSPC transplantation into the post-ischemic environment may lead to graft overgrowth and tumor formation due to their pluripotency and ability to self-renew [67]. While VEGF infusion may look promising, injection of recombinant human VEGF has been shown to promote blood vessel permeability as well as blood-brain barrier leakage [43]. Thus, elucidating the mechanisms by which NSPCs protect the ischemic penumbra following stroke will provide novel therapeutic strategies for facilitating the contribution of endogenous NSPCs for brain repair.

## Methods

### Primary cortical neuronal and neural stem/progenitor cultures

Ethics Statement: This study was approved by the University of New Mexico Animal Care and Use Committee and conformed to the NIH Guidelines for use of animals in research. Timed pregnant female mice were sacrificed by isoflurane overdose and the embryos removed by cesarean section. Primary neuronal cultures were established from cerebral cortices of C57BL/6J embryos (The Jackson Laboratory, Bar Harbor, ME, USA) at gestation day 15, using enzymatic dissociation with trypsin as previously described [37]. For immunocytochemistry, 3.9×10^5^ dissociated cells were plated on poly-L-lysine-coated coverslips (0.1 mg/ml; Sigma, St Louis, MO, USA) in 24-well plates. For biochemical procedures, 1.95×10^6^ cells were plated on precoated poly-L-lysine six-well plates (BD Biosciences, San Diego, CA, USA). The cultures were maintained under serum-free conditions in neurobasal medium (Invitrogen Corp., Carlsbad, CA, USA), supplemented with B-27 supplement (2%; Invitrogen Corp.), glutamine (0.5 mM; Sigma), glutamate (25 *µ*M; Sigma), penicillin (100 U/ml) and streptomycin (100 *µ*g/ml; Invitrogen Corp.). At 4 days *in vitro* (DIV), half of the medium was removed and replaced with fresh medium without glutamate, as indicated by the manufacturer. At 7 DIV, neuronal cultures were used for experimentation. Embryonic neural stem/progenitor cells (eNSPC) were established from whole telencephalon of EGFP-C57BL/6J embryos at gestation day 14 (E14), as described previously [68]. Postnatal NSPCs (pNSPC) were established from postnatal day 28 male EGFP-C57BL/6J mice. NSPCs were plated on pre-coated poly-L-lysine six-well plates (BD Biosciences). Growth factors were added every other day and cells were passaged at least twice prior to use. NSPCs used for coculture were plated on 0.4 µM pore size transwells (BD Biosciences) coated with poly-L-lysine (0.1 mg/mL; Sigma). The cultures were maintained under serum-free conditions in neurobasal medium (Invitrogen Corp.), supplemented with B-27 supplement (2%; Invitrogen Corp.), glutamine (2.0 mM; Sigma), penicillin (100 U/ml; Invitrogen Corp.), streptomycin (100 *µ*g/ml; Invitrogen Corp.), EGF (10 ng/mL; Invitrogen Corp.), and bFGF (10 ng/mL; Invitrogen Corp.). All cultures were maintained in a humidified incubator at 37°C with 5% CO_2_. The cultures were incubated with the following compounds: SU1498 (70 nM–50 *µ*M; Calbiochem, San Diego, CA, USA), IgG-Fc (5 ng/mL-1 µg/mL; Jackson ImmunoResearch Laboratories, West Grove, PA, USA), and Flt-1-Fc (5 ng/mL-1 µg/mL; R&D Systems, Minneapolis, MN, USA).

### Oxygen–glucose deprivation

Oxygen-glucose deprivation (OGD) was performed as previously described [36], [37]. Primary cortical neuronal cultures and/or NSPCs were placed in an anaerobic chamber (Coy Laboratories, Grass Lake, MI, USA) containing a gas mixture of 5% CO_2_, 5% H_2_ and 85% N_2_ (<0.2% O_2_). Normal culture media was replaced with deoxygenated, glucose-free Earle's balanced salt solution, and the cultures were maintained under glucose-free anaerobic conditions at 37°C for 2 h. OGD was terminated by returning the cultures to normoxic conditions and neurobasal medium supplemented with 2% B-27 supplement, 0.5 mM glutamine, 100 U/ml penicillin and 100 *µ*g/ml streptomycin. As a control, sister cultures were placed in Earle's balanced salt solution containing 25 mM glucose (Sigma) for 2 h at 37°C under normoxic (21% O_2_) conditions and then returned under normoxic conditions to neurobasal medium supplemented with 2% B-27 supplement, 0.5 mM glutamine, 100 U/ml penicillin and 100 *µ*g/ml streptomycin.

### Mild transient middle cerebral artery occlusion (MCAO)

Adult male C57BL/6J mice 20–25 g (The Jackson Laboratory) were housed under a 12 h light: 12 h dark cycle with food and water available *ad libitum*. Mice (20–25 g; n = 20) were anesthetized with 4% isoflurane for induction and maintained on 1.0% isoflurane. The body temperature was maintained at 37°C with a heating pad. Mice were subjected to 30 minutes of transient MCAO as previously described [69]. Briefly, the right common carotid artery was exposed through a midline incision. The internal carotid artery was isolated from external carotid artery which was ligated with 6–0 silk suture. A 2-cm length of 6–0 rounded tip nylon suture was advanced from the common carotid artery (CCA), through the internal carotid artery up to the level of the anterior cerebral artery. The suture was inserted 10 to 11 mm from the bifurcation of CCA to occlude the middle cerebral artery (MCA). After 30 min of MCAO, the suture was slowly withdrawn to allow for reperfusion. Reperfusion time was 3 days post-MCAO. The mice were anesthetized with sodium pentobarbital (150 mg/kg, Fort Dodge Animal Health, Fort Dodge, IA, USA) administered intraperitoneally, and were transcardially perfused first with preperfusing solution (0.01 mg/ml heparin and 1 mg/ml procaine) and subsequently with 4% paraformaldehyde in 0.1 M phosphate buffered saline (PBS). The brains were removed, post-fixed overnight in 4% paraformaldehyde, and cryoprotected with 30% sucrose (w/v) in 0.1 M PBS for 48 hours at 4°C. For immunohistochemical procedures (described below), the brains were sectioned at 30 µm using a sliding microtome (AO Scientific Instruments, Buffalo, NY, USA).

### Embryonic NSPC Transplantation

NSPCs isolated from E14 EGFP-expressing transgenic C57BL/6J mouse embryos were expanded in culture as described above and transplanted into 8 week-old C57BL/6J mouse striatum. Mice were anaesthetized with 2% isoflurane inhalation. Stereotaxic injections were performed using Hamilton microsyringe with a 26-gauge blunt needle. Each animal received an injection of 2.5 µl (at the rate of 1 µl/min, and concentration 5×10^4^ cells/µl) of EGFP-NSPCs into the striatum (from bregma: A +1.0 mm, L +2.0 mm, V −2.6 mm). To identify fragmentation of DNA characteristic of apoptosis, TUNEL labeling was performed on paraformaldehyde fixed tissue from 30 minutes MCAO using NeurotacsTM II kit (Trevigen, Inc, Gaithersburg, MD, USA), according to the manufacturer's protocol. Tissue treated with TACs-nuclease™ or without terminal deoxynucleotidyl transferase served as positive and negative controls, respectively. NIH ImageJ software was used to quantify TUNEL+ cells from fluorescent images using Sholl analysis, with concentric rings placed at increasing distances (635 µm apart) around the perimeter of the graft.

### Immunohistochemistry

Cultured cells were fixed on coverslips with 4% paraformaldehyde and incubated in monoclonal mouse anti-Nestin (1∶500; Chemicon, Temecula, CA, USA), polyclonal rabbit anti-MAP-2 (1∶500; Chemicon), and/or polyclonal rabbit anti-GFAP (1∶500; Accurate, Westbury, NY, USA). Histological sections (30 µm) were incubated in monoclonal mouse anti-NeuN (1∶1000, Chemicon) or polyclonal rabbit anti-MPO (1∶500, Santa Cruz Biotechnology, Santa Cruz, CA, USA). Immunofluorescence was visualized using FITC- or Cy3-conjugated secondary antibodies (1∶250; Jackson ImmunoResearch Laboratories). DAPI nuclear stain was used to identify cell bodies of cultured cells (Invitrogen Corp.). Immunofluorescence was analyzed using high-resolution confocal microscopy (Zeiss LSM510, Thornwood, NY, USA) or conventional fluorescence microscopy.

### Immunoassays

Soluble VEGF or BDNF were assayed from conditioned media using the Quantikine Mouse Immunoassay for the appropriate protein (R&D Systems). HIF-1α was measured in cell lysates using Surveyor™ IC Human/Mouse Total HIF-1α Immunoassay (R&D Systems). Transcriptional activity of HIF-1α was measured in nuclear extract using the TransAM HIF-1 kit (Active Motif, Carlsbad, CA, USA), substituting the primary antibody for one that recognizes mouse (Novus Biologicals, Littleton, CO, USA). Nuclear extracts used in this assay were isolated using the NE-PER Nuclear and Cytoplasmic Extraction Reagent Kit (Pierce, Rockford, IL, USA). Absorbance for all assays was read using a microplate reader (Dynex Technologies, Chantilly, VA, USA): Quantikine assays were read at 450 nm with background subtraction of 570 nm; TransAM assay was read at 450 nm with a background subtraction of 630 nm.

### MTT cell viability assay

Cell viability was assessed using methylthiazolyldiphenyl-tetrazolium bromide (MTT) dissolved in PBS (0.5 mg/mL; Sigma). Cultures were incubated with MTT at 37°C for 4 hr. Formazan crystals were solubilized using 1∶1 ethanol:DMSO solution. Absorbance was measured at 570 nm with a background subtraction of 630 nm.

### HIF-1α gene deletion from NSPCs

NSPCs were isolated from embryonic day 14 transgenic mice containing loxP-flanked exon 2 of the HIF-1α gene (HIF-1α^fl/fl^) [38] that were crossed to the C57BL/6J background (The Jackson Laboratory). NSPCs were passaged twice prior to 48 hr treatment with an adenovirus expressing Cre-recombinase (Ad-CMV-Cre; Vector Biolabs, Philadelphia, PA, USA) at 50 MOI (multiplicity of infection). This method recombines the DNA between the two loxP sites to excise the intervening sequence. Twenty-four hours after infection, NSPCs were passaged twice more before used in experiments. To confirm deletion of exon 2 of the HIF-1α gene, DNA was isolated and efficiency of cre-recombination was detected with PCR using forward primer 5′- TGT TAA ATA AAA GCT TGG AC -3′ and reverse primer 5′- GCA GTT AAG AGC ACT AGT TG -3′ [70]. Successful DNA recombination yields HIF-1α gene-deleted NSPCs (HIF-1α^Δ/Δ^), as indicated by the loss of the 1200 bp band amplification product and appearance of a 250 bp band. Experiments using HIF-1α^Δ/Δ^ eNSPCs were compared to wild-type C57BL/6J eNSPCs treated similarly with Ad-CMV-Cre.

### Statistical Analysis

Data are expressed as means ± S.E.M. Significant differences between means were determined by student's t-test or analysis of variance (ANOVA) with Tukey multiple comparisons *post hoc* analysis using Prism software (Graphpad Software, San Diego, CA, USA). P values < 0.05 are considered statistically significant.

## Supporting Information

Figure S1 Myeloperoxidase (MPO) staining for activated neutrophils. (A–D) Myeloperoxidase (MPO) staining of coronal histological sections through the ischemic striatum 3 days following MCAO. Mice received intrastriatal injections of exogenous EGFP+NSPCs (A–C) or PBS (D) 72 hr prior to MCAO. Scale bar: 10 µm.
(4.33 MB TIF)

Figure S2 Dose-response of neurons to SU1498 and Flt-1-Fc. Neurons were exposed to increasing concentrations of the VEGFR2 inhibitor SU1498 (A) or the decoy VEGFR1 Flt-1-Fc (B). SU1498 was cytotoxic at concentrations of 50 and 100 µM, but not at 10 µM which was the dose used in our experiments (A). A toxic dose of Flt-1-Fc was not determined (B).
(0.22 MB TIF)

Figure S3 NSPC response to inhibition of autocrine VEGF. Embryonic NSPCs were exposed to the VEGFR1 decoy Flt-1-Fc (1 µg/mL). Flt-1-Fc did not impair the viability of eNSPCs, and did not impair their ability to survive OGD.
(0.16 MB TIF)

Figure S4 NSPC viability in response to 2 hr OGD. Deletion of exon 2 of the HIF-1α gene did not impair eNSPC ability to survive 2 hr OGD, as compared to wild-type eNSPCs.
(0.14 MB TIF)
