# Supplementary figures and images for: Murine Neural Stem/Progenitor Cells Protect Neurons against Ischemia by HIF-1α–Regulated VEGF Signaling
Source: PLoS One. 2010 Mar 22;5(3):e9767. doi: 10.1371/journal.pone.0009767 (PMC2842303; doi:10.1371/journal.pone.0009767)

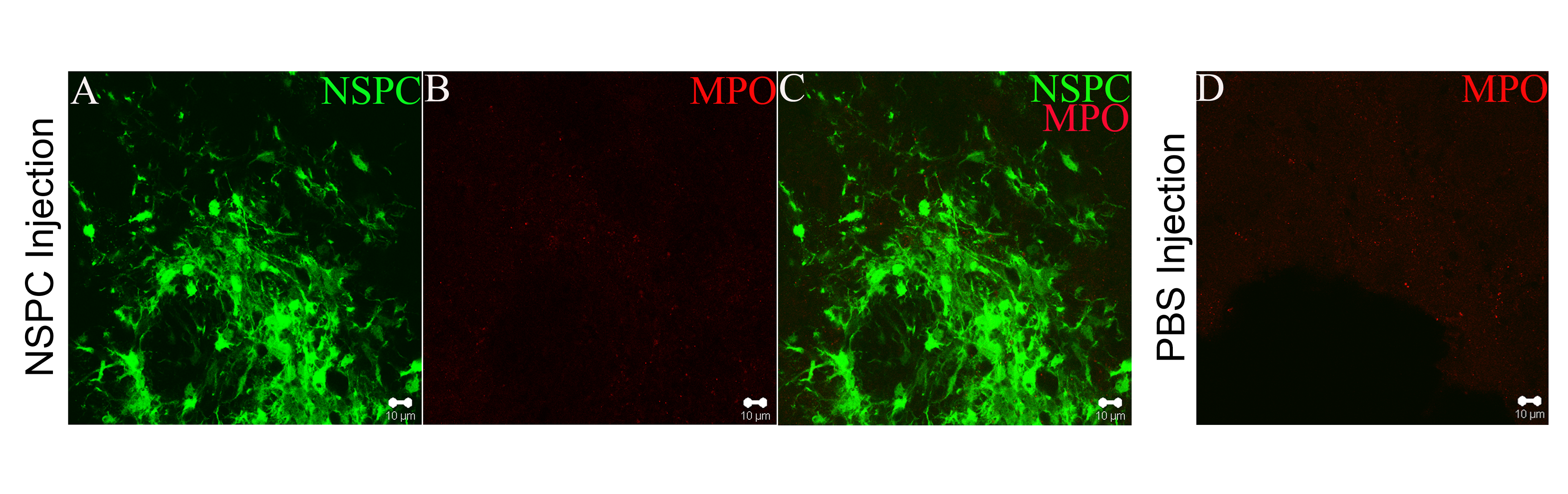

Supplement: Figure S1 — Myeloperoxidase (MPO) staining for activated neutrophils. (A–D) Myeloperoxidase (MPO) staining of coronal histological sections through the ischemic striatum 3 days following MCAO. Mice received intrastriatal injections of exogenous EGFP+NSPCs (A–C) or PBS (D) 72 hr prior to MCAO. Scale bar: 10 µm. (4.33 MB TIF) [file pone.0009767.s001.tif]

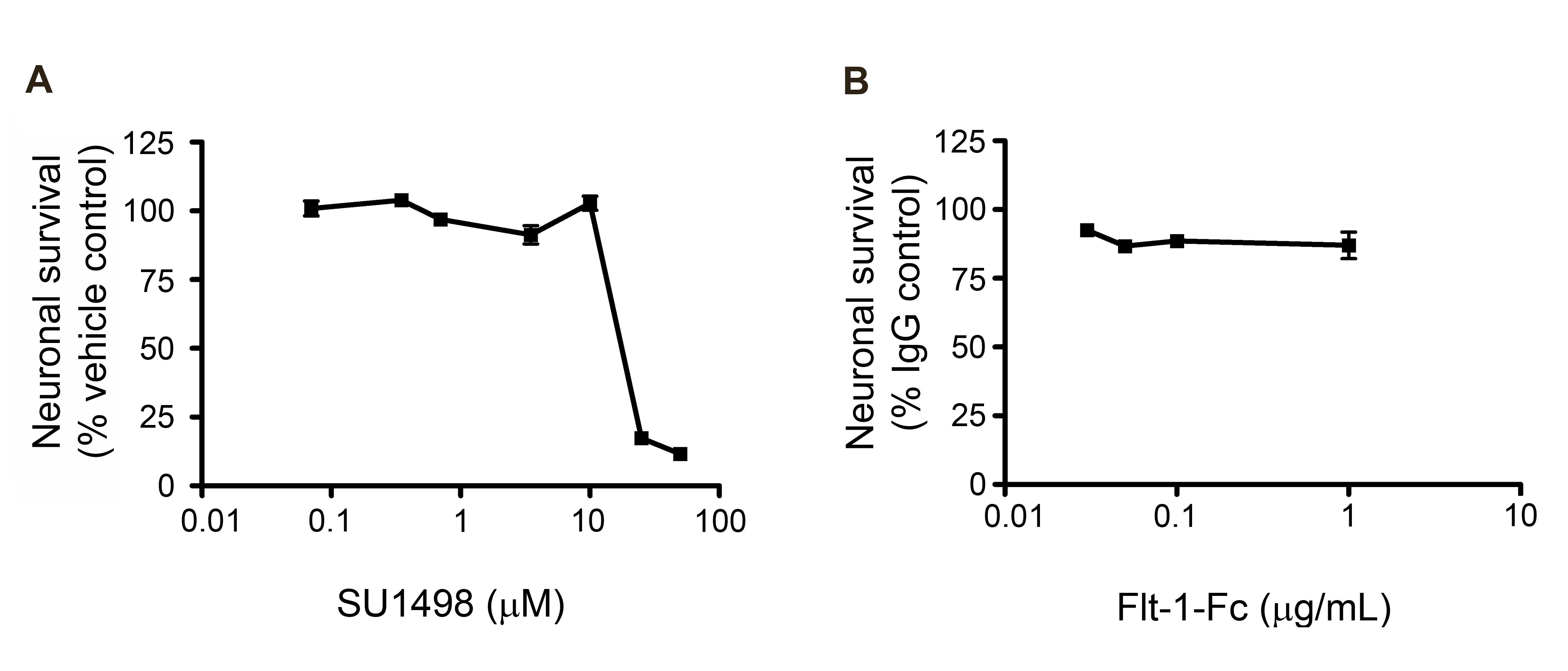

Supplement: Figure S2 — Dose-response of neurons to SU1498 and Flt-1-Fc. Neurons were exposed to increasing concentrations of the VEGFR2 inhibitor SU1498 (A) or the decoy VEGFR1 Flt-1-Fc (B). SU1498 was cytotoxic at concentrations of 50 and 100 µM, but not at 10 µM which was the dose used in our experiments (A). A toxic dose of Flt-1-Fc was not determined (B). (0.22 MB TIF) [file pone.0009767.s002.tif]

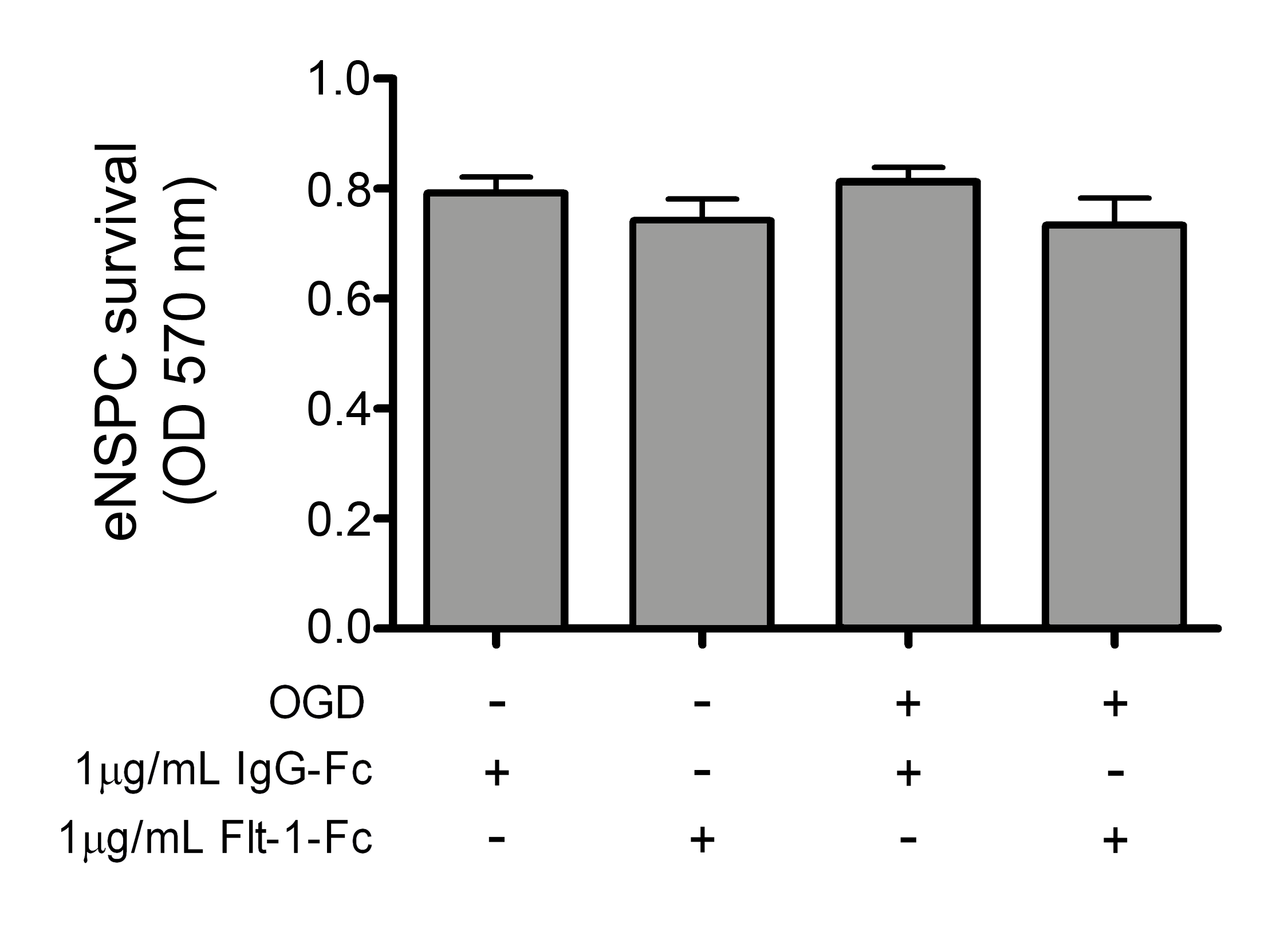

Supplement: Figure S3 — NSPC response to inhibition of autocrine VEGF. Embryonic NSPCs were exposed to the VEGFR1 decoy Flt-1-Fc (1 µg/mL). Flt-1-Fc did not impair the viability of eNSPCs, and did not impair their ability to survive OGD. (0.16 MB TIF) [file pone.0009767.s003.tif]

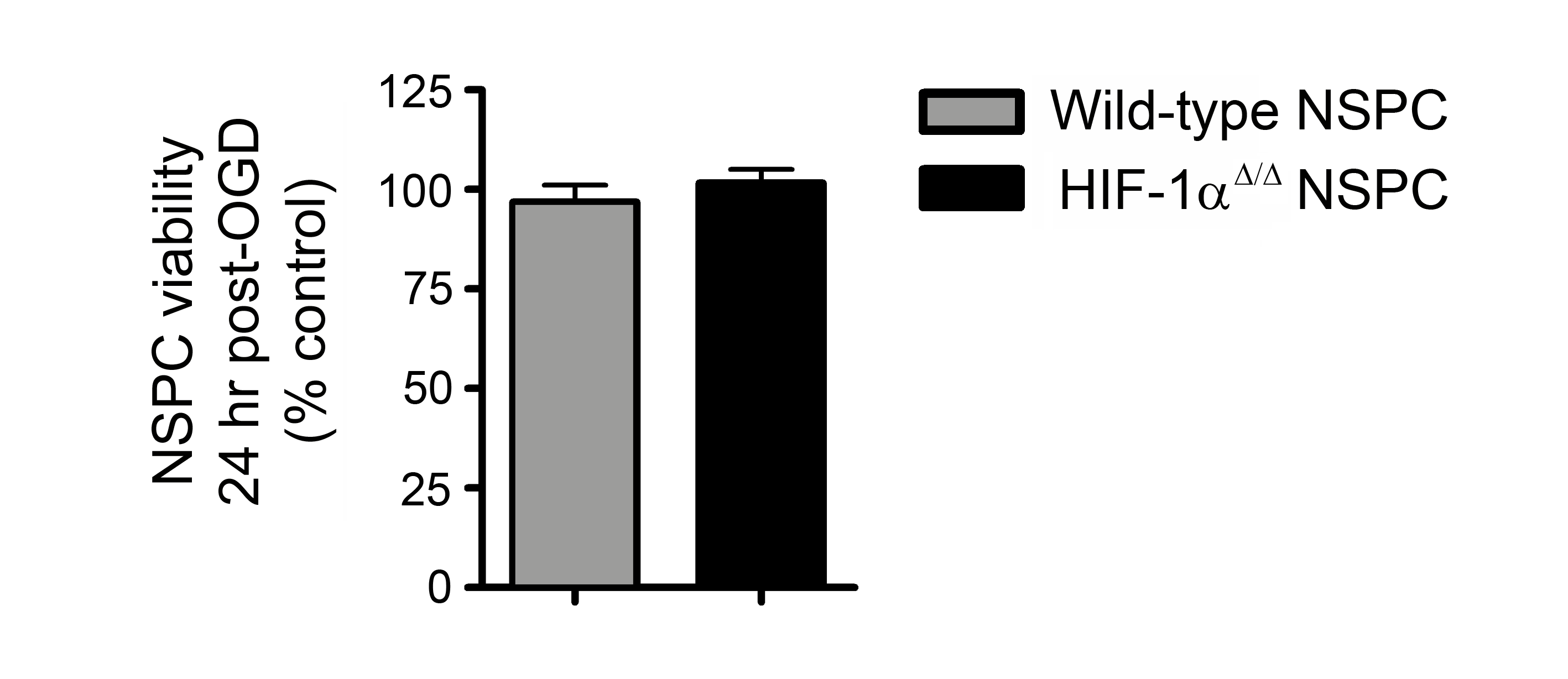

Supplement: Figure S4 — NSPC viability in response to 2 hr OGD. Deletion of exon 2 of the HIF-1α gene did not impair eNSPC ability to survive 2 hr OGD, as compared to wild-type eNSPCs. (0.14 MB TIF) [file pone.0009767.s004.tif]
